# Phosphocitrate Is Potentially a Disease-Modifying Drug for Noncrystal-Associated Osteoarthritis

**DOI:** 10.1155/2013/326267

**Published:** 2013-02-21

**Authors:** Yubo Sun, David R. Mauerhan, Atiya M. Franklin, James Norton, Edward N. Hanley, Helen E. Gruber

**Affiliations:** ^1^Department of Orthopedic Surgery, Carolinas Medical Center, P.O. Box 32861, Charlotte, NC 28232, USA; ^2^Department of Biostatistics, Carolinas Medical Center, P.O. Box 32861, Charlotte, NC 28232, USA

## Abstract

Phosphocitrate (PC), a calcification inhibitor, inhibits the development of crystal-associated osteoarthritis (OA) in Hartley guinea pigs. However, the molecular mechanisms underlying its disease-modifying effect remain elusive. This study sought to test the hypothesis that PC has calcium crystal-independent biological activities which are, at least in part, responsible for its disease-modifying activity. We found that PC inhibited the proliferation of OA fibroblast-like synoviocytes in the absence of calcium crystals. Consistent with its effect on cell proliferation, PC downregulated the expression of numerous genes classified in cell proliferation. PC also downregulated the expression of many genes classified in angiogenesis and inflammatory response including prostaglandin-endoperoxide synthase 2, interleukin-1 receptor, type I, and chemokine (C-C motif) ligand 2. In contrast, PC upregulated the expression of many genes classified in musculoskeletal tissue development, including aggrecan, type I collagen, and insulin-like growth factor binding protein 5. These findings suggest that PC is not only a promising disease-modifying drug for crystal-associated OA but also for noncrystal-associated OA.

## 1. Introduction 

Osteoarthritis (OA) is a progressive disorder characterized by the degeneration of articular cartilage, formation of osteophytes, and synovial lining hyperplasia. It is one of the most prevalent causes of disability in the aging population and has enormous economic and social consequences. However, existing treatment options only provide symptomatic relief and have no effect on the progression of the underlying disease. The lack of progress in the development of disease-modifying drugs for OA therapy is largely due to our limited understanding of the pathogenesis of the disease and our insufficient knowledge about the molecular targets for intervention. 

The most apparent pathological changes in OA are usually found in articular cartilage. Synovium of patients with OA has traditionally been considered to be normal and is used as control in studies to investigate the pathological changes in the synovium of patients with rheumatoid arthritis (RA) [1, 2]. OA fibroblast-like synoviocytes (FLSs) have also been used as control cells in some studies [3, 4]. However, synovial lining hyperplasia and inflammation, a potential leading cause of knee pain, are common findings in OA patients [5, 6]. Studies have demonstrated that OA synovium and OA FLSs display different gene expression profiles compared with the synovium and FLSs derived from normal control subjects [7, 8]. These findings indicate that OA synovium plays an important role in the pathogenesis of OA.

Basic calcium phosphate (BCP) crystal and calcium pyrophosphate dihydrate (CPPD) crystal are the two most common intra-articular crystals [9, 10]. The presence of these crystals in the synovial fluid or joint tissues of end-stage OA patients is a well-recognized event [11–14]. In cell culture, calcium crystals induced mitogenesis, production of matrix metalloproteinases (MMPs), and endocytotic activity of cells [15–18]. However, the clinical significance of these calcium crystals and their role in the development and/or progression of OA remain unclear [14, 19–21]. 

Phosphocitrate (PC), a potent anticalcification molecule, was originally identified in mammalian mitochondria [22]. PC inhibits the formation and growth of calcium crystals by its strong binding to amorphous calcium phosphates aggregates and the surface of calcium crystals. In cell cultures, PC inhibited crystal-induced mitogenesis, expression of MMPs, and cell death [23–25]. In Hartley guinea pig model of crystal-associated OA, PC inhibited meniscal calcification and reduced the degeneration of articular cartilage [26]. These findings appear to provide support for the notions that calcium crystals play an important role in the development and/or progression of OA and that calcification inhibitors are promising disease-modifying drugs for crystal-associated OA therapy [27]. However, a bisphosphonate, a potent calcification inhibitor similar to PC, failed to inhibit the development of OA in Hartley guinea pigs, raising questions about the role of crystals in the development of crystal-associated OA [28]. Several studies indicate that PC has biological activities unrelated to its anticalcification activity. For example, PC reduced the degradation of low-density lipoprotein by 60% [29]. PC inhibited transforming growth factor-*β*1-induced proliferation of progressive ankylosis fibroblasts [30]. We decide to perform this study to test the hypothesis that PC has unique crystal-independent biological activities which are responsible, at least in part, for its disease-modifying activity. 

## 2. Materials and Methods

Dulbecco's modified eagle medium (DMEM), fetal bovine serum (FBS), and stock antibiotic/antimycotic mixture were products of Invitrogen (Carlsbad, CA, USA). PC was synthesized according to the procedure previously described [31]. 

### 2.1. Cell Cultures

Telomerase-transduced human OA FLSs, or hTERT-OA 13A FLSs, have been previously described [8]. Primary OA FLSs were prepared from synovial tissues collected with the approval of the authors' institutional review board from OA patients undergoing total joint replacement surgery at our medical center. The need for informed consent was waived because the synovial tissues were surgical waste, and no private patient information was collected. Briefly, synovial tissues were minced into small pieces (3 mm × 3 mm) and cultured in 100 mm plates at 37°C in DMEM containing 10% FBS. Every three days, culture medium was changed. When OA FLSs reached 80% confluence, they were harvested and passaged. Human foreskin fibroblasts were obtained from ATCC (CRL-2429, Mananas, USA) and expanded in DMEM containing 10% FBS. 

#### 2.1.1. Cell Proliferation

hTERT-OA 13A FLSs (4 × 10^4^) were plated in six-well cluster plates. On the second day, DMEM containing 10% FBS and PC was added to the top three wells. DMEM containing 10% FBS without PC was added to bottom three wells. The culture medium was changed every three days until the cells in the bottom wells without PC reached 85% confluence. All cells were then harvested and cell numbers were determined by cell count using a hemocytometer. This proliferation assay was also performed using primary OA FLSs and foreskin fibroblasts for comparison.

### 2.2. RNA Extraction

hTERT-OA 13A FLSs were plated in four 100 mm plates at 90% confluence. On the second day, DMEM containing 1% FBS was added. On the third day, DMEM in two plates was replaced with DMEM containing 1% FBS and PC at a final concentration of 0.6 mM. DMEM in other two plates was replaced with DMEM containing 1% FBS but without PC. Twenty-four hours later, total RNA was extracted from these cells using Trizol reagent (Invitrogen, Carlsbad, CA, USA) and purified using Oligotex kit (Qiagen, Valencia, CA, USA). These RNA samples were used for microarray analyses and RT-PCR experiments.

### 2.3. Microarray

Double-stranded DNA was synthesized using SuperScript double-stranded cDNA synthesis kit (Invitrogen, Carlsbad, CA, USA). The DNA product was purified using GeneChip sample cleanup module (Affymetrix, Santa Clara, CA, USA). cRNA was synthesized and biotin labeled using BioArray high yield RNA transcript labeling kit (Enzo Life Sciences, Farmingdale, NY, USA). The cRNA product was purified using GeneChip sample cleanup module and subsequently chemically fragmented. The fragmented, biotinylated cRNA was hybridized to HG-U133_Plus_2 gene chip using Affymetrix Fluidics Station 400 (Affymetrix, Santa Clara, CA, USA). The fluorescent signals were quantified during two scans by Agilent Gene Array Scanner G2500A (Agilent Technologies, Palo Alto, CA, USA) and GeneChip operating Software (Affymetrix, Santa Clara, CA, USA). Genesifter software (VizX Labs, Seattle, WA, USA) was used for the analysis of differential gene expression and gene ontology.

### 2.4. Real-Time RT-PCR

Briefly, cDNA was synthesized using TaqMan Reverse Transcription Reagents (Applied Biosystems, Inc., University Park, IL, USA) using the RNA samples described. Quantification of relative transcript levels of selected genes was performed using ABI7000 Real-Time PCR system (Applied Biosystems, Inc., University Park, IL, USA). TaqMan Gene Expression assays (Applied Biosystems, Inc., University Park, IL, USA) were used. cDNA samples were amplified with an initial Taq DNA polymerase activation step at 95°C for 10 minutes, followed by 40 cycles of denaturation at 95°C for 15 seconds and annealing at 60°C for one minute. Fold change was calculated, and the expression level of the genes was normalized to the expression level of the housekeeping gene glyceraldehyde 3-phosphate dehydrogenase (GAPDH) according to the method described [32]. The experiments were performed independently using RNA samples extracted from both hTERT-OA 13A FLSs and primary OA FLSs.

### 2.5. Statistical Analysis

Data are expressed as the mean ± SD. For cell proliferation, the difference between two experimental groups was analyzed using Student's *t*-test. For real-time RT-PCR, experiment was repeated twice in triplicates. The difference between two experimental groups was analyzed using Student's *t*-test. In all cases, *P* values less than 0.01 were considered significant. Statistical analysis was performed using the SAS software, version 9.3.

## 3. Results

### 3.1. PC Inhibited Proliferation of OA FLSs

**Figure 1:**
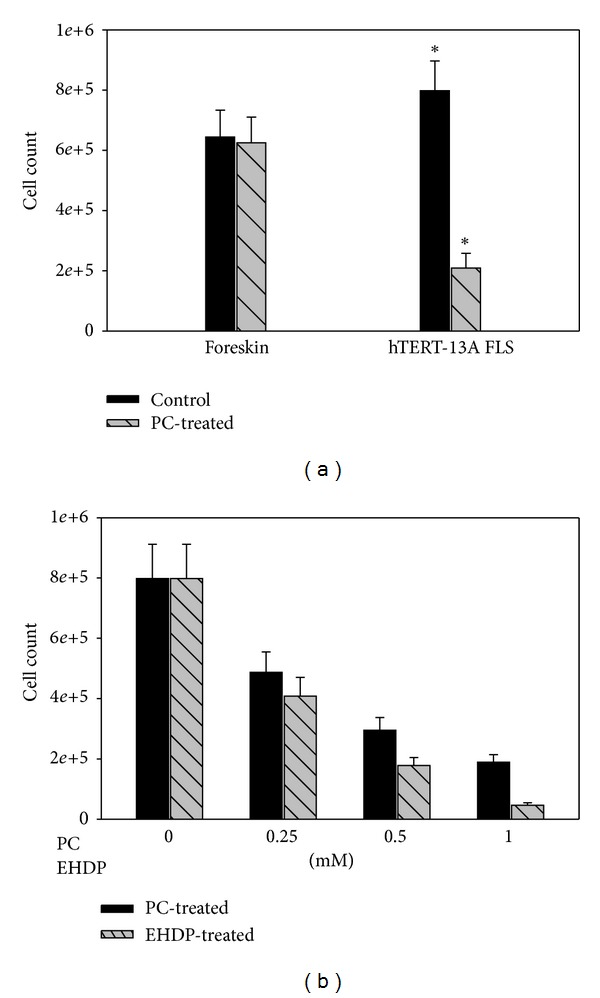
PC inhibits the proliferation of OA FLSs. (a)There were about 65% fewer hTERT-OA 13A FLSs in the PC-treated wells after nine days of culture compared to those in the untreated wells (*P* < 0.01). (b) PC and EHDP inhibited the proliferation of primary OA FLSs in a dose-dependent manner.

 PC had no effect on the proliferation of human foreskin fibroblasts (Figure 1(a), the left bar group). However, PC inhibited the proliferation of hTERT-OA 13A FLSs (Figure 1(a), the right bar group). There were about 60% fewer hTERT-OA 13A FLSs in the PC-treated wells after nine days of culture compared with the cells in the untreated wells (*P* < 0.01). 

Next, we examined the effect of PC on cell proliferation using primary OA FLSs. For comparison, we also examined the effect of disodium salt of ethane-1-hydroxy-1, 1-bisphosphonic acid (EHDP), which is a bisphosphonate. As shown in Figure 1(b), both PC and EHDP inhibited the proliferation of primary OA FLSs in a dose-dependent manner. The morphologies of PC-treated OA FLSs and untreated OA FLSs were similar (not shown), indicating that the reduction in cell number was not due to cellular toxicity of PC.

### 3.2. Effect of PC on Gene Expression

Microarray analysis revealed that of the more than 50,000 transcripts examined, 3,011 transcripts displayed significant differential expression (more than 1.5-fold) between the PC-treated hTERT-OA 13A FLSs and the untreated hTERT-OA 13A FLSs; 1,558 transcripts were downregulated, and 1,453 transcripts were upregulated by PC. Differentially expressed genes were classified according to gene ontology category biological process. The genes that fell into specific biological processes previously implicated in OA, or suspected to have a role in OA are listed in Tables 1 and 2. 

As shown in Table 1, the expression of numerous genes classified in cell proliferation was downregulated by PC. Of the 32 differentially expressed genes with more than twofold changes between the PC-treated cells and untreated cells, the expression of 30 genes, including cyclin E1 (CCNE1; −2.30-fold change), cyclin E2 (CCNE2; −3.74-fold change), and cell division cycle 25 homolog C (CDC25C; −2.31-fold change), was downregulated by PC. This downregulatory effect of PC on the genes associated with cell proliferation is consistent with the finding that PC inhibited the proliferation of OA FLSs (Figure 1).

The expression of many genes classified in angiogenesis and inflammatory response was also downregulated by PC. Of the 18 differentially expressed genes classified in angiogenesis, the expression of 13 genes, including neuropilin 1 (NRP1; −2.69-fold change) and TEK tyrosine kinase (TEK; −2.58-fold change), was downregulated by PC. Of the 22 differentially expressed genes classified in inflammatory response, the expression of 15 genes, including prostaglandin-endoperoxide synthase 2/cyclooxygenase (PTGS2/Cox-2; −6.09-fold change), chemokine (C-C motif) ligand 2 (CCL2/MCP-1; −1.82-fold change), and chemokine (C-X-C) ligand 2 (CXCL2; 1.66-fold change), was downregulated by PC. In addition, of the 3 differentially expressed genes classified in response to pain, the expression of all 3 genes, including tachykinin receptor 1 (TACR1, −4.39), was downregulated by PC. Of the 5 differentially expressed genes classified in the response to interleukin-1 (IL-1), the expression of 4 genes, including V-src sarcoma viral oncogene homolog (SRC, −3.95-fold change), IL-1 receptor type 1 (IL1R1; −1.59-fold change), and IL-1 receptor-associated kinase 2 (IRAK2; −1.82-fold change), was downregulated by PC. 

Furthermore, the expression of many genes classified in the cytokine- and chemokine-mediated signal pathway and IL-6 production was downregulated by PC (Table 1). Of the 9 differentially expressed genes classified in the cytokine and chemokine-mediated signal pathway, the expression of 7 genes, including chemokine (C-C motif) receptor 1 (CCR1, −1.60-fold change), was downregulated by PC. Of the 7 differentially expressed genes classified in IL-6 production, the expression of 6 genes, including tumor necrosis factor, alpha-induced protein 3 (TNFAIP3, −2.07-fold), was downregulated by PC. 

Most of the genes upregulated by PC fell into biological processes associated with musculoskeletal tissue development. For example, of the 25 differentially expressed genes classified in muscle tissue development, the expression of 15 genes, including insulin-like growth factor binding protein 5 (IGFBP5; 8.57-fold change), was upregulated by PC. Of the 28 differentially expressed genes classified in skeletal development, the expression of 18 genes, including annexin A2 (2.17-fold change), vitamin D (1,25- dihydroxyvitamin D3) receptor (VDR; 2.11-fold change), aggrecan (ACAN; 1.80-fold change), collagen type 1, alpha-1 (COL1A1; 1.66-fold change), and collagen type XI alpha-1 (COL11A1; 1.50-fold change), was upregulated by PC (Table 2). 

### 3.3. Real-Time RT-PCR

The genes selected for validation for the differential expression between the PC-treated and untreated hTERT-OA 13A FLSs by real-time RT-PCR were listed in Table 3. As shown, the differential expressions of the genes examined were confirmed by real-time RT-PCR.

## 4. Discussion

Previous studies demonstrated that PC inhibited BCP crystal-stimulated mitogenesis and expression of PTGS2/Cox-2 [15, 33, 34]. These observations provided the bases for the hypothesis that PC is potentially a disease-modifying drug for crystal-associated OA. However, the findings presented in this study clearly indicate that the inhibitory activities of PC on mitogenesis and proliferation of OA FLSs and the downregulatory activity of PC on the expression of PTGS2/Cox-2 are intrinsic properties of PC. These distinct biological activities of PC are not dependent on the presence of calcium crystals. 

Synovial hyperplasia is associated with the severity of knee pain and fast hyaline articular cartilage loss in OA [35, 36]. Synovial hyperplasia is also associated with synovial angiogenesis [37]. OA FLSs, but not normal control FLSs, induced a significant enhancement of angiogenesis [38]. These observations indicate that OA FLSs play an important role in the pathogenesis of OA. In this study, we demonstrated that PC not only downregulated the expression of numerous genes associated with cell proliferation but also downregulated the expression of many genes associated with angiogenesis. These findings indicate that PC is potentially an antisynovial hyperplasia agent.

PTGS2/Cox-2 is a molecular target for the management of arthritis pain and inflammation [39, 40]. The strong downregulation of PTGS2/Cox-2 by PC suggests that PC may have some effect on the pain associated with OA. This potential effect of PC on pain is further supported by the finding that PC significantly downregulated the expression of TACR1 (−4.39-fold change) and SRC (−3.95-fold change) (Table 1). TACR1 is a G protein-coupled receptor which is upregulated in the joint tissues of patients with painful OA [41]. SRC, a tyrosine kinase, plays a role in substance P signaling which has been implicated in many inflammatory diseases [42, 43]. These findings together indicate that PC is potentially an analgesic agent.

In this study, we demonstrated that PC strongly downregulated the expression of many genes classified in cytokine- and chemokine-mediated signal pathway, including IL1R1, CCL2, and CCR1. IL-1*β* is a main inflammatory cytokine found within OA joints and represents one of the possible treatment targets. CCL2 is a small chemokine which recruits leukocytes to synovial inflammation site and is central to the development of pain and inflammation associated with OA [44]. The findings presented in this study indicate that PC may inhibit inflammatory cell activation and infiltration and that PC is potentially an anti-inflammator agent.

We also demonstrated that PC downregulated the expression of plasminogen activator, urokinase (PLAU, −2.21-fold change), PLAU receptor (PLAUR, −1.92-fold change), early growth response 1 (EGR-1, −2.25-fold change), v-fos FBJ murine osteosarcoma viral oncogene homolog (FOS, −1.65-fold change), and FOS-like antigen 1 (FOSL1, −1.87-fold change). PLAU is a serine protease which catalyzes the conversion of inactive zymogen plasminogen to active protease plasmin when binding to its receptor PLAUR. Plasmin is capable of degrading all the components of the extracellular matrix and activating other enzymes such as MMPs. Higher levels of PLAU and PLAUR were detected in arthritic specimens [45–47]. EGR-1, FOS, and FOSL1 are involved in FLSs activation and implicated in the pathogenesis of RA [48–50]. The downregulation of these genes by PC supports the hypothesis that PC is potentially a disease-modifying drug for OA therapy. 

Interestingly, PC upregulated the expression of many genes classified in muscle and skeletal tissue development, including IGFBP5. Previous studies demonstrated that inhibition of IGFBP5 proteolysis improved the joint architecture and reduced articular cartilage loss in a dog model of OA [51–53]. The strong upregulation of IGFBP5 by PC is clearly consistent with the notion that PC is potentially a disease-modifying drug for OA therapy. Moreover, PC upregulated the expression of ACAN (1.80-fold change), COL1A1 (1.66-fold change), COL1A2 (1.88-fold change), COL3A1 (1.83-fold change), COL11A1 (1.50-fold change), and COL12A2 (1.93-fold change), indicating that PC may improve the integrity of synovial tissues.

Surprisingly, PC upregulated the expression of MMP3 (3.18-fold change) and ADAM metallopeptidase with thrombospondin type 1 motif 5 (ADAMTS5; 3.32-fold change) (Table 2). The upregulation of both extracellular matrix proteins and extracellular matrix protein-degrading enzymes by PC indicates that PC may promote synovial tissue repair and regeneration. The implication of this specific effect on the disease process of OA is unclear at present. 

## 5. Conclusions

PC is potentially an antisynovial hyperplasia, analgesic and anti-inflammator agent. PC is not only a promising disease-modifying drug for crystal-associated OA but also a promising disease-modifying drug for noncrystal-associated OA. The findings presented in this study provide further support for the development of PC, and/or its analogues, as disease-modifying drugs for OA therapy. 

## Figures and Tables

**Table 1:** Differentially expressed genes in PC-treated hTERT-OA 13A FLS compared with the untreated cells.

Biological process	Gene name	Gene ID	Differ. expre. (fold)*	Description
Cell proliferation	BLM	NM_000057	−3.64	Bloom syndrome
	CCNE2	AF112857	−3.74	Cyclin E2
	CCNE1	AI671049	−2.30	Cyclin E1
	CDC25A	AY137580	−3.63	Cell division cycle 25 homolog A (*S. pombe*)
	CDC25C	NM_001790	−2.31	Cell division cycle 25 homolog C (*S. pombe*)
	CDC2	AA749427	−3.13	Cell division cycle 2, G1 to S and G2 to M
	CDC6	NM_001254	−2.36	Cell division cycle 6 homolog (*S. cerevisiae*)
	CDC7	NM_003503	−2.12	Cell division cycle 7 homolog (*S. cerevisiae*)
	CDCA5	BE614410	−2.41	Cell division cycle associated 5
	CDCA8	BC001651	−2.11	Cell division cycle associated 8
	CDK2	AB012305	−2.74	Cyclin-dependent kinase 2
	NCAPH	D38553	−2.64	Non-SMC condensin I complex, subunit H
	HELLS	NM_018063	−2.49	Helicase, lymphoid specific
	AURKB	AB011446	−2.43	Aurora kinase B
	KIF23	AW192521	−2.41	Kinesin family member 23
	CLASP2	BC029035	−2.40	Cytoplasmic linker-associated protein 2
	NUF2	AF326731	−2.35	NUF2, NDC80 kinetochore complex component, homolog
	DSN1	NM_024918	−2.35	DSN1, MIND kinetochore complex component, homolog
	SPC24	AI469788	−2.32	SPC24, NDC80 kinetochore complex component, homolog
	SPC25	AF225416	−2.10	SPC25, NDC80 kinetochore complex component, homolog
	HMGA2	AI990940	−2.30	High mobility group AT-hook 2
	LIG1	NM_000234	−2.25	Ligase I, DNA, ATP-dependent
	KIFC1	BC000712	−2.21	Kinesin family member C1
	BRCA2	X95152	−2.18	Breast cancer 2, early onset
	ERCC6L	NM_017669	−2.17	Excision repair cross-complement repair deficiency, comp. group 6 like
	SPAG5	NM_006461	−2.16	Sperm-associated antigen 5
	NEK2	Z25425	−2.14	Never-in-mitosis-gene-A- (NIMA) related kinase 2
	NCAPG	NM_022346	−2.12	Non-SMC condensin I complex, subunit G
	ZWINT	NM_007057	−2.01	ZW10 interactor antisense
	PARD3B	AF428251	3.24	Par-3 partitioning defective 3 homolog B (*C. elegans*)
	11-Sep	AI333326	2.28	Septin 11

Angiogenesis	NRP1	AF280547	−2.69	Neuropilin 1
	TEK	BF594294	−2.58	TEK tyrosine kinase, endothelial
	ELK3	NM_005230	−2.42	ELK3, ETS-domain protein (SRF accessory protein 2)
	EREG	NM_001432	−1.90	Epiregulin
	PML	AW291023	−1.89	Promyelocytic leukemia
	COL15A1	NM_001855	−1.80	Collagen, type XV, alpha-1
	NRP2	AI819729	−1.75	Neuropilin 2
	SPHK1	NM_021972	−1.72	Sphingosine kinase 1
	FOXC2	NM_005251	−1.68	Forkhead box C2, mesenchyme forkhead 1 (MFH-1)
	SCG2	NM_003469	−1.66	Secretogranin II (chromogranin C)
	EDNRA	NM_001957	−1.56	Endothelin receptor type A
	TGFBR2	NM_003242	−1.51	Transforming growth factor, beta-receptor II (70/80kDa)
	ROBO4	AA156022	−1.51	Roundabout homolog 4, magic roundabout (*Drosophila*)
	JAG1	AI457817	2.42	Jagged 1 (Alagille syndrome)
	NOTCH4	AI341271	1.75	Notch homolog 4 (Drosophila)
	RUNX1	D89788	1.73	Runt-related transcription factor 1
	EPAS1	NM_001430	1.67	Endothelial PAS domain protein 1

Inflammatory response	PTGS2	AY151286	−6.09	Prostaglandin-endoperoxide synthase 2
	SERPINA1	AF119873	−2.15	Serpin peptidase inhibitor, clade A
	GPR68	AI805006	−2.15	G protein-coupled receptor 68
	BMPR1B	AA935461	−2.12	Bone morphogenetic protein receptor, type IB
	EVI1	BE466525	−2.00	Ecotropic viral integration site 1
	FOS	BC004490	−1.92	V-fos FBJ murine osteosarcoma viral oncogene homolog
	IRAK2	AI246590	−1.82	Interleukin-1 receptor-associated kinase 2
	CCL2	S69738	−1.82	Chemokine (C-C motif) ligand 2
	CCR1	NM_001295	−1.60	Chemokine (C-C motif) receptor 1
	CXCL2	M57731	−1.66	Chemokine (C-X-C motif) ligand 2
	SPN	BC035510	−1.79	Sialophorin (leukosialin, CD43)
	TLR4	AF177765	−1.70	Toll-like receptor 4
	SCG2	NM_003469	−1.66	Secretogranin II (chromogranin C)
	FN1	AJ276395	−1.58	Fibronectin 1
	KLKB1	BE326857	−1.52	Cytochrome P450, family 4, subfamily V, polypeptide 2
	NDST1	NM_001543	2.05	N-Deacetylase/N-sulfotransferase (heparan glucosaminyl) 1
	C3	NM_000064	2.05	Complement component 3
	SERPINA3	NM_001085	1.88	Serpin peptidase inhibitor, clade A
	SBNO2	AC005390	1.78	Strawberry notch homolog 2 (Drosophila)
	NFKBIZ	BE646573	1.74	Nuclear factor of kappa light polypeptide enhancer in B-cells inhibitor zeta
	MASP1	NM_001879	1.64	Mannan-binding lectin serine peptidase 1
	STAT5B	NM_012448	1.59	Signal transducer and activator of transcription 5B

Response to pain	TACR1	AA461490	−4.39	Tachykinin receptor 1
	COMT	BG149428	−2.03	Catechol-O-methyltransferase
	CACNA1A	BC042451	−2.00	Calcium channel, voltage-dependent, P/Q type, alpha-1A subunit

Response to interleukin-1	SRC	NM_005417	−3.95	V-src sarcoma viral oncogene homolog (avian)
	IRAK2	AI246590	−1.82	Interleukin-1 receptor-associated kinase 2
	PCSK1	NM_000439	−1.60	Proprotein convertase subtilisin/kexin type 1
	IL1R1	AK026803	−1.59	Interleukin-1 receptor, type I
	GHR	NM_000163	1.76	Growth hormone receptor

Cytokine- and chemokine- mediated	EREG	NM_001432	−1.90	Epiregulin
	CCL2	S69738	−1.82	Chemokine (C-C motif) ligand 2
	LIFR	NM_002310	−1.77	Leukemia inhibitory factor receptor-alpha
	RQCD1	BC007102	−1.76	RCD1 required for cell differentiation1 homolog (S. pombe)
	LRP8	NM_004631	−1.73	Low-density lipoprotein receptor-related protein 8, apolipoprotein e receptor
	CCR1	NM_001295	−1.60	Chemokine (C-C motif) receptor 1
	IL1RI	AK026803	−1.59	Interleukin-1 receptor, type I
	STAT3	BF508977	2.01	Signal transducer and activator of transcription 3
	STAT5B	NM_012448	1.59	Signal transducer and activator of transcription 5B

Interleukin-6 production	BCL10	AA994334	−2.38	B-cell CLL/lymphoma 10
	TNFAIP3	AI738896	−2.07	Tumor necrosis factor, alpha-induced protein 3
	FOXP3	NM_014009	−1.92	Forkhead box P3
	EREG	NM_001432	−1.90	Epiregulin
	TOLR4	AF177765	−1.70	Toll-like receptor 4
	ADORA2B	NM_000676	−1.59	Adenosine A2b receptor
	IL6R	NM_000565	2.03	Interleukin-6 receptor

Miscellaneous	PLAU	K03226	−2.21	Plasminogen activator, urokinase
	PLAUR	U08839	−1.92	Plasminogen activator, urokinase receptor
	PLA2G4A	M68874	−1.51	Phospholipase A2, group IVA (cytosolic, calcium dependent)
	PTGER4	AA897516	−1.91	Prostaglandin E receptor 4 (subtype EP4)
	EGR1	AI459194	−2.25	Early growth response 1
	FOS	BC004490	−1.92	V-fos FBJ murine osteosarcoma viral oncogene homolog
	FOSL1	BG251266	−1.95	FOS-like antigen 1
	THBS1	BF084105	2.41	Thrombospondin 1

*Negative number indicates decreased expression (fold) in PC-treated hTERT-OA 13A FLS compared with the untreated cells. Positive number indicates elevated expression (fold) in PC-treated hTERT-OA 13A FLS compared with the untreated cells.

**Table 2:** Differentially expressed genes in PC-treated hTERT-OA 13A FLS compared to the untreated cells.

Biological process	Gene name	Gene ID	Differ. Expre. (fold)*	Description
Muscle tissue development	IGFBP5	AW157548	8.57	Insulin-like growth factor binding protein 5
	CACNB4	NM_000726	2.73	Calcium channel, voltage-dependent, beta-4-subunit
	TPM1	AI521618	2.43	Tropomyosin 1 (alpha)
	JAG1	U61276	2.02	Jagged 1 (Alagille syndrome)
	MORF4L2	H43976	1.90	Mortality factor 4 like 2
	NRG1	NM_013957	1.88	Neuregulin 1
	SIRT2	BG722779	1.86	Sirtuin (silent mating type information regulation 2 homolog) 2
	NF1	D12625	1.80	Neurofibromin 1
	OBSL1	BF446688	1.78	Obscurin-like 1
	MBNL1	AA732240	1.73	Muscleblind-like (Drosophila)
	TPM1	NM_000366	1.72	Tropomyosin 1 (alpha)
	CAV2	AA150110	1.67	Caveolin 2
	RXRA	BE675800	1.66	Retinoid X receptor, alpha
	NR2F2	AL554245	1.63	Nuclear receptor subfamily 2, group F, member 2
	TCF7L2	AV721430	1.61	Transcription factor 7-like 2 (T-cell specific, HMG-box)
	TBX2	U28049	−4.17	T-box 2
	ADRB2	NM_000024	−2.36	Adrenergic, beta-2-receptor, surface
	SORT1	BE742268	−1.93	Sortilin 1
	GJC1	NM_005497	−1.77	Gap junction protein, gamma 1, 45 kDa
	CENPF	U30872	−1.77	Centromere protein F, 350/400 ka (mitosin)
	BCL2	NM_000657	−1.71	B-cell CLL/lymphoma 2
	TBX3	U69556	−1.71	T-box 3
	SDC1	NM_002997	−1.65	Syndecan 1
	TBX5	AW269421	−1.54	T-box 5
	RARB	NM_015854	−1.51	Retinoic acid receptor, beta

Skeletal development	ANXA2	D28364	2.17	Annexin A2
	VDR	AA772285	2.11	Vitamin D (1,25-dihydroxyvitamin D3) receptor
	GNAS	AI693143	1.95	GNAS complex locus
	ACAN	NM_001135	1.80	Aggrecan
	COL1A1	AI743621	1.66	Collagen, type I, alpha-1
	COL1A2	AA628535	1.88	Collagen, type I, alpha-2
	COL11A1	NM_001854	1.50	Collagen, type XI, alpha-1
	COL12A1	AU146651	1.93	Collagen, type XII, alpha-1
	MSX2	D89377	1.85	Msh homeobox 2
	GHR	NM_000163	1.76	Growth hormone receptor
	MEF2C	AL536517	1.59	Myocyte enhancer factor 2C
	THRA	NM_003250	1.57	Thyroid hormone receptor, alpha
	RUNX2	AW469546	1.55	Runt-related transcription factor 2
	CLEC3B	NM_003278	1.55	Exosome component 7
	MEF2C	N22468	1.55	Myocyte enhancer factor 2C
	IGFBP4	NM_001552	1.54	Insulin-like growth factor binding protein 4
	PRKRA	AA279462	1.53	Protein kinase, interferon-inducible RNA-dependent activator
	TNFRSF11B	NM_002546	1.50	Tumor necrosis factor receptor superfamily, member 11b
	BMPR1B	AA935461	−2.12	Bone morphogenetic protein receptor, type IB
	ANKH	AF274753	−1.93	Ankylosis, progressive homolog (mouse)
	ACVR2A	NM_001616	−1.89	Activin A receptor, type IIA
	CYTL1	NM_018659	−1.83	Cytokine-like 1
	TBX3	U69556	−1.71	T-box 3 (ulnar mammary syndrome)
	SOX9	NM_000346	−1.71	SRY- (sex-determining-region-Y-) box 9
	FOXC2	NM_005251	−1.68	Forkhead box C2 (MFH-1, mesenchyme forkhead 1)
	KIAA1217	BC017424	−1.66	KIAA1217
	MMP9	NM_004994	−1.61	Matrix metallopeptidase 9
	TGFBR2	NM_003242	−1.51	Transforming growth factor, beta-receptor II (70/80 kDa)

Collagen biosynthetic process	COL3A1	AU146808	1.83	Collagen, type III, alpha-1
	COL1A2	AA628535	1.88	Collagen, type I, alpha-2
	COL1A1	AI743621	1.66	Collagen, type I, alpha-1
	TRAM2	AI986461	1.58	Translocation-associated membrane protein 2
	ITGA2	N95414	−1.74	Integrin, alpha-2 (CD49B, alpha-2 subunit of VLA-2 receptor)

Collagen catabolic process	MMP3	NM_002422	3.18	Matrix metallopeptidase 3 (stromelysin 1, progelatinase)
	ADAMTS5	BI254089	3.32	ADAM metallopeptidase with thrombospondin type 1 motif, 5
	ADAMTS14	W60649	2.15	ADAM metallopeptidase with thrombospondin type 1 motif, 14
	MMP16	U79292	1.80	Matrix metallopeptidase 16 (membrane inserted)
	MMP9	NM_004994	−1.61	Matrix metallopeptidase 9

*Negative number indicates decreased expression (fold) in PC-treated hTERT-OA 13A FLS compared with the untreated cells. Positive number indicates elevated expression (fold) in PC-treated hTERT-OA 13A FLS compared with the untreated cells.

**Table 3:** Differential expression confirmed by real-time RT-PCR.

Gene name	Gene ID	Differential expression microarray	Differential expression RT-PCR*	Differential expression RT-PCR**
BLM	NM_000057	−3.64	−4.23	−3.95
HELLS	NM_018063	−2.49	−2.87	−2.12
CCNE2	AF112857	−3.74	−3.93	−3.70
CDC25C	NM_001790	−2.31	−2.01	−1.94
EGR1	AI459194	−2.25	−2.45	−3.29
FOSL1	BG251266	−1.95	−2.31	−2.11
PLAUR	U08839	−1.92	−2.41	−3.22
PLA2G4A	M68874	−1.51	−1.86	−2.11
PTGS2	AY151286	−6.09	−5.77	−5.10
TACR1	AA461490	−4.39	−3.92	−3.45
CCL2	S69738	−1.82	−2.02	−1.79
GPR68	AI805006	−2.15	−2.54	−2.28
CACNA1A	BC042451	−2.00	−2.10	−2.22
IGFBP5	AW157548	8.57	6.32	5.46
ADAMTS5	BI254089	3.32	2.81	2.11
COL1A2	AA628535	1.88	1.40	1.64

*Differential expression—the numbers are the ratio of the relative expression level of a specific gene in PC-treated hTERT-OA 13A FLSs compared with the relative expression level of the specific gene in the untreated hTERT-OA 13A FLSs. **Differential expression—the numbers are the ratio of the relative expression level of a specific gene in PC-treated primary FLSs compared with the relative expression level of the specific gene in the untreated primary OA FLSs.
